# A166 STEROID-REFRACTORY ILEITIS FOLLOWING RITUXIMAB THERAPY: A CASE REPORT AND LITERATURE REVIEW

**DOI:** 10.1093/jcag/gwad061.166

**Published:** 2024-02-14

**Authors:** A Alalool, S Lee

**Affiliations:** Department of Medicine, University of Toronto, Toronto, ON, Canada; Department of Medicine, University of Toronto, Toronto, ON, Canada

## Abstract

**Background:**

Rituximab, an anti-CD20 monoclonal antibody commonly used in treatment of hematologic and rheumatologic disease is generally well-tolerated from a gastrointestinal perspective however rare reported side effects include diarrhea and enterocolitis.

**Aims:**

We describe a case of steroid-refractory ileitis presenting in a woman with follicular lymphoma post Rituximab therapy.

**Methods:**

A case report and literature review were performed.

**Results:**

A 73-year-old woman presented with a 4-week history of fevers, bloody diarrhea and abdominal pain. She had a known history of stage IV follicular lymphoma treated with six cycles of Bendamustine and Rituximab followed by four doses of maintenance Rituximab, with the last dose ending 4 weeks prior to her presentation. On examination, she had multiple peri-anal round ulcers. An abdominal CT revealed circumferential mural thickening involving the distal terminal ileum. On colonoscopy, there were multiple discrete ulcers and nodularity in the terminal ileum. Pathology showed evidence of chronic active ileitis with no signs of malignancy, lymphoproliferative disorders, or granulomas. AFB smear and cultures were negative. She was given IV steroids for 1 week and then placed on a Prednisone taper scheduled to finish in 9 weeks.

4 weeks later she presented again with fevers and abdominal pain. Inflammatory markers were elevated, and a repeat CT abdomen showed ongoing multifocal ileitis. She was put back on IV steroids and monitored. She continued to have persistent abdominal pain and fevers, and then underwent a repeat colonoscopy after 9 days of IV steroids which showed an interval increase in the size of her ileal ulcers. Pathology showed granulation tissue and reactive changes with no evidence of malignancy, lymphoma, granulomas, inclusion bodies or other viral cytopathic effects. Repeat AFB smear was negative. CMV PCR in blood was negative. She was assessed for Behcet’s disease (BD) given her peri-anal ulcers however she lacked other findings needed to fulfill the diagnostic criteria for BD. Steroids were continued with plans to initiate biological therapy.

A review of literature revealed rare reports of rituximab-associated ileocolitis and development of de novo inflammatory bowel disease post rituximab therapy. Among patients who received Rituximab and underwent Colonoscopy, 4% had evidence of colitis. Rituximab-associated colitis appears to be usually mild presenting with diarrhea and requiring supportive care only, but in rare cases can lead to fulminant colitis and bowel perforation.

**Conclusions:**

Rituximab-associated enterocolitis remains an under-reported entity and can lead to significant morbidity and treatment-associated challenges requiring prolonged courses of steroids and considerations for biological therapies. Important differentials to consider include malignancy, lymphoma, Tuberculosis, Bechet’s disease, and CMV enterocolitis.

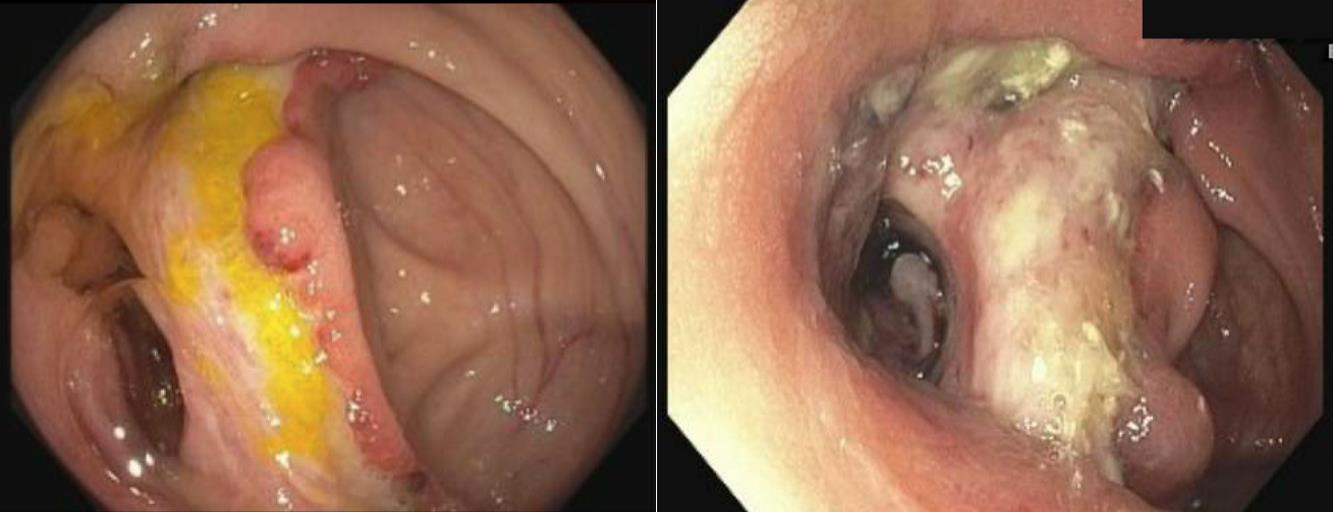

Ulcer in terminal ileum and ileocecal valve - Pre steroids (Left), Post steroids (Right)

**Funding Agencies:**

None

